# Use of participatory visual narrative methods to explore older adults’ experiences of managing multiple chronic conditions during care transitions

**DOI:** 10.1186/s12913-018-3292-6

**Published:** 2018-06-20

**Authors:** Chantal Backman, Dawn Stacey, Michelle Crick, Danielle Cho-Young, Patricia B. Marck

**Affiliations:** 1 School of Nursing, Faculty of Health Sciences, University of Ottawa, 451, Smyth Rd, RGN 3239, Ottawa, ON K1H 8M5 Canada; 2 Ottawa Hospital Research Institute, 451, Smyth Road, Ottawa, ON K1H 8M5 Canada; 3 Faculty of Human and Social Development, University of Victoria, PO Box 1700, STN CSC, Victoria, BC V8W 2Y2 Canada

**Keywords:** Patient engagement, Person- and family-centred care, Patient safety, Patient experience, Complex care, Visual methods

## Abstract

**Background:**

Older adults with multiple chronic conditions typically have more complex care needs that require multiple transitions between healthcare settings. Poor care transitions often lead to fragmentation in care, decreased quality of care, and increased adverse events. Emerging research recommends the strong need to engage patients and families to improve the quality of their care. However, there are gaps in evidence on the most effective approaches for fully engaging patients/clients and families in their transitional care. The purpose of this study was to engage older adults with multiple chronic conditions and their family members in the detailed exploration of their experiences during transitions across health care settings and identify potential areas for future interventions.

**Methods:**

This was a qualitative study using participatory visual narrative methods informed by a socio-ecological perspective. Narrated photo walkabouts were conducted with older adults and family members (*n* = 4 older adults alone, *n* = 3 family members alone, and *n* = 2 older adult/family member together) between February and September 2016. The data analysis of the transcripts consisted of an iterative process until consensus on the coding and analysis was reached.

**Results:**

A common emerging theme was that older adults and their family members identified the importance of active involvement in managing their own care transitions. Other themes included positive experiences during care transitions; accessing community services and resources; as well as challenges with follow-up care. Participants also felt a lack of meaningful engagement during discharge planning, and they also identified the presence of systemic barriers in care transitions.

**Conclusion:**

The results contribute to our understanding that person- and family-centered care transitions should focus on the need for active involvement of older adults and their families in managing care transitions. Based on the results, three areas for improvement specific to older adults managing chronic conditions during care transitions emerged: strengthening support for person- and family-centered care, engaging older adults and families in their care transitions, and providing better support and resources.

## Background

With the population rapidly aging, the global burden of chronic diseases is also increasing dramatically [1, 2]. Approximately three quarters of older adults in Canada reported having at least one chronic health condition [3]. Older adults with multiple chronic conditions typically have more complex needs, and often seek care across different healthcare settings [4]. A care transition is defined as the movement of a patient between different healthcare settings, and/or between health care providers [5]. In addition, these older adults may have multiple care transitions between health settings (e.g., acute care to convalescence to home) [6]. However, these older adults rarely have cohesive treatment plans due to the lack of coordination amongst different health care providers [7]. Poor care transitions often lead to fragmentation in care, decreased quality of care, and an increase in adverse events [8–11]. An Ontario study examined the experiences of older adults and their caregivers with navigating through the Canadian health care system, and found that 66% of participants reported difficulties [12]. Research has attributed these long-standing difficulties to the inadequate engagement of patients and families in the transitional care [13–15].

Engaging with patients and/or their family members is fundamental to a person-centered approach; and it is also key to improving overall care and controlling costs in our health care system [16]. Person-centred care incorporates the person’s unique needs, values and preferences into their treatment plan [17]. It may also encompass the complete spectrum of engagement, from simply understanding the person’s experiences and perspectives with the health system to fully involving them in improving their own health and the health of their communities [16, 18]. Research evidence indicates that people who are more involved in the decision-making process related to their care are better able to manage complex chronic conditions [17, 19, 20], and have shorter lengths of stay in hospital [21]. Shared decision-making ensures that the patient is better supported, better informed and more encouraged to participate in their health care plans [22]. This has been shown to result in more effective and trusting relationships between patients/clients and clinicians [23] with better adherence to treatment recommendations [24].

However, there are gaps in evidence on the most effective approaches for fully engaging patients/clients and families in their transitional care. We also do not know in what context this engagement can be sustained across the health care system – specifically, for older adults with multiple chronic conditions who tend to have the most care transitions. The purpose of this study was to engage older adults with multiple chronic conditions and their family members in the detailed exploration of their experiences during transitions across health care settings and identify potential areas for future interventions.

## Methods

### Research design and methodology

We conducted a descriptive qualitative study using participatory visual narrative methods, which consisted of older adult/family narrated photo walkabouts***.*** A walkabout is a guided ‘ecological tour’ of the environment to tap into the knowledge that older adults have about the different places where they receive care in order to understand their experience [25]. The purpose of the photo walkabouts was to enhance our ability to capture ‘visual’ experiences instead of only verbal events during their transitions. The photographs are a powerful tool to facilitate participants’ ability to articulate their own stories (photo narration) and generated robust dialogue on issues and potential solutions for safer care transitions. Photo narration is a way of surfacing participants’ stories, history, and culture in relation to the photographs [25]. This methodology was selected to actively engage patients and families in the research process.

Ethical approval was obtained from the University of Ottawa Research Ethics Board.

### Theoretical perspective

These methods were informed by a socio-ecological perspective, and adapted from previous research in health care [26, 27]. This perspective incorporates knowledge from a broader context including social, economic, political, and cultural factors [28]. An integrative systems approach to studying health system issues using a socio-ecological or whole systems perspective [29, 30] supports the notion of patients and health care providers working together to adaptively manage and mitigate challenges in their health care environment [26, 27].

### Setting and participants

Older adults and families were recruited from the community home care provider organization in a suburban area using convenience sampling. The inclusion criteria were older adults: managing at least two chronic conditions (e.g. chronic obstructive pulmonary disease, congestive heart failure, diabetes, coronary artery disease, and stroke); receiving primary care services for greater than 90 days; who experienced at least one transfer across sectors within the past 90 days (e.g., from acute care to home or from rehab to home, etc.); aged 50 years or older (men and women); able to communicate in English; and able to sign consent. Family members who were at least 18 years of age or older were also invited to participate.

### Procedure and data collection

The care coordinator of the home care provider organization helped identify the potential participants that met the eligibility criteria and helped to introduce the study. The trained research assistant (MC) informed the interested participants of the study researchers background and professional affiliations and provided an explanation of the study guided by the participant information sheet. The research assistant confirmed their interest and willingness to participate, and obtained their written consent. After obtaining consent, the trained research assistant conducted 60 to 90-min audio-recorded photo walkabout sessions in the older adults’ preferred location. All participants identified their home as their preferred location for the photo walkabouts, but participants were asked to share their experience of care transitions across health care settings, and not only their experiences in the home environment. The walkabout was led by the research assistant. The aim of these discussions were to generate meaningful dialogue and knowledge exchange on the participants’ overall experiences during care transitions.

### Data analysis

Three research team members (MC, DC, and CB) independently reviewed and coded each transcript. The results from individual analyses were then collectively analyzed by these three team members for similarities across the transcripts using an iterative process until consensus on the coding and analysis was reached [31]. ATLAS.ti (Scientific Software Development GmBH, Berlin) was used to manage the visual and textual data and supported the thematic analysis of the photo walkabout narratives. Two other team members (DS, PBM) contributed to the team’s discussion of the findings. Participants were not offered to review and comment on the findings. This study adhered to the Consolidated Criteria for Reporting Qualitative Research checklist for reporting qualitative research [32].

## Results

We recruited a total of nine study participants (*n* = 4 older adults alone, *n* = 3 family members alone, and *n* = 2 older adult/family member together) between February and September 2016. Participants were recruited until no new data was obtained from the walkabout narratives. Three participants were registered with and receiving services from a Health Link, which is defined as: “a team of providers in a geographic area (i.e., primary care, hospital, home, community care, long-term care providers community support agencies, and other community partners) working together to provide coordinated health care to patients with multiple complex conditions” [33]. Six participants were receiving home care services through a home care service agency. Together, the nine participants experienced a total of 22 different transitions within the last two years (see Table 1).

**Table 1:** Participant demographic information

Older adults	n
Older adults receiving home services	9
Health Link	3
Home care service agency	6
Mean age (range) in years	77.6 (56–94)
Marital status	
Married	3
Single / Widowed	6
Living arrangements	
Alone	3
With spouse	3
With other family	2
Assisted Living	1
Gender	
Female	6
Male	3
Family members/informal caregivers	n
Older adults with caregiver/family member support	8
Relationship to older adult	
Spouse	3
Daughter	5
Gender (caregiver)	
Female	7
Male	1

Older adults and their families were actively engaged in describing their care transition experiences. Six key themes were identified through the narratives and photographs, and are discussed below. Participants were identified by participant type and number (i.e., P1 = patient number 1; F7 = family member number 7; PF4 = patient and family member number 4).

### Theme 1: Active involvement in care transitions

Active involvement in care transitions included sub-themes of partners in care planning, families as critical advocates, getting organized for self-monitoring, and acting upon previous experiences.

#### Partners in care planning

Participants discussed the importance of being involved in verifying discharge medications; organizing or re-establishing community services; maintaining a copy of medical records; and alerting family doctors of any concerns following discharge. PF4 described their active involvement in their mother’s care: *“…. I’m not leaving till I go through all the previous medication, make sure they’ve been transcribed again, new one is 20 ml prescription says 15 ml, can you please get the doctor to fix this…I say I’m not leaving until it’s corrected.”* PF4 was also able to guide the team in the hospital, with regard to post discharge services for their mother *“because we had experience with things…and asked because we knew it [was] a fabulous facility, could she go to rehab for a week… in terms of transitions between hospital and home it made a huge difference.”* P2 explained*“… I have to be watching all of the time.”*

#### Families as critical advocates

Families advocated for their family member and helped with navigating the healthcare system during the transition. PF4 described her responsibility as a family member, to work in partnership with the health system to avoid her mother “falling through the cracks” between services. She said: *“their list of priorities is long and mine is one. So I understand that. But my mother’s not going to fall through the cracks because they have other priorities.”* PF4 felt it was the role of families to be an advocate for their loved one: *“People have to understand [that] in any transition, any hospital stay, [the healthcare professionals] do a fabulous job, the resources are stretched. The family has got to be there; the family has got to be engaged.”* PF5 described her daughter as well informed about health care resources available and using her knowledge of the health system to navigate the transition between services. *“One of my daughters works as a medical secretary in the hospital and so she’s, what we call, the air traffic controller. She really knows how to get everything going…she’ll problem solve a lot of things on the side. So I kind of [take care of] the health side. She does the kind of operational things that need organization.”*

#### Getting organized for self-monitoring

**Fig. 1:**
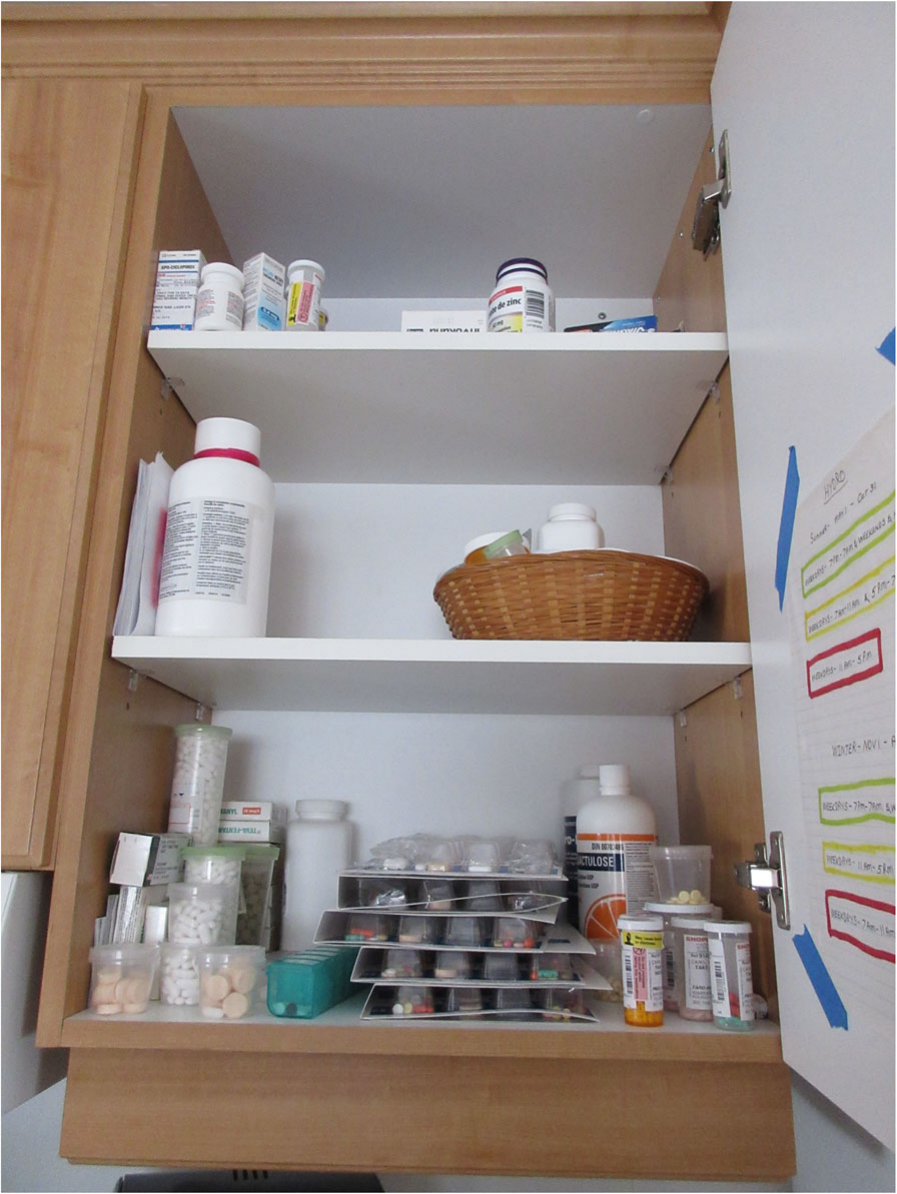
Medication cabinet

**Fig. 2:**
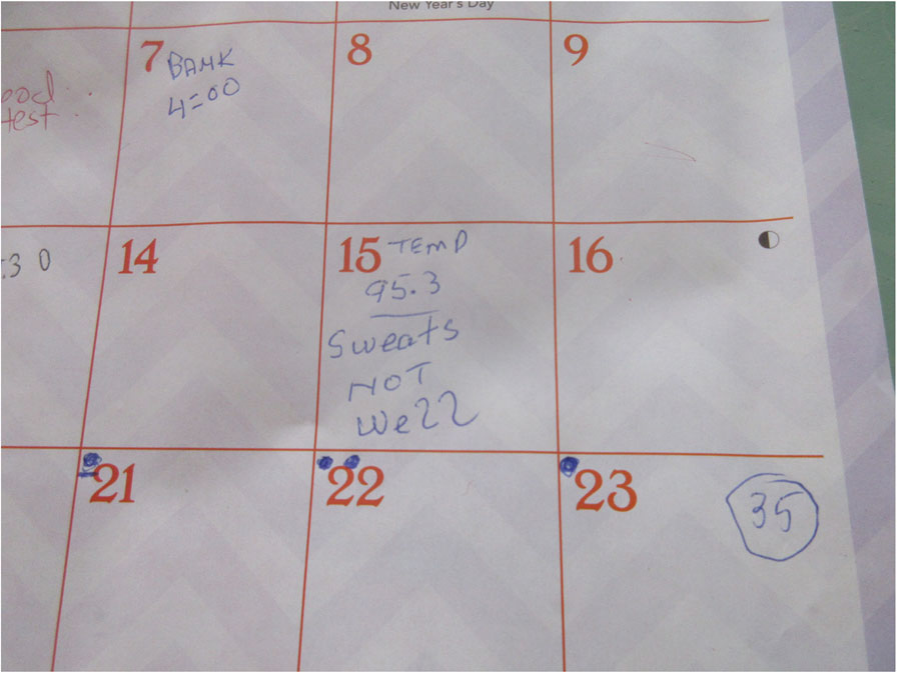
‘Tracking’ schedule for symptom management, and for follow-up appointments

P2 showed her medication cabinet (Fig. 1) and said: *“the medications are accurate because I [have] a dosette. Don’t think I’m not a sick person, cause this is my cabinet...”* She also showed how she keeps track of her signs and symptoms (Fig. 2), and explained: *“So this is why now I’m starting to write on a calendar days that I don’t feel good. I want to catch it the day that I’m not feeling good. So, I will give you an example: During the night, I took my temperature. I was hypothermic. I was putting it on my calendar and I was thinking I could feel the cold blood. I thought it was the thermometer, so I went and bought a new thermometer, started doing the test again and the thermometer was right both times. So, it was obviously me who wasn’t feeling good and the thermometer was right. The doctor phoned, and I did the blood test that afternoon. The results came back and the doctor said I was dehydrated. So obviously there was a problem there. But we still haven’t found out why.”*

#### Acting upon previous experiences

PF4 described that they were able to better navigate the system based on their previous experiences. *“Again it’s through experience, I know as soon as she goes into the hospital, [the home care services] get informed and the services stop. OK, the stress begins close to that two week cut off period, because after two weeks you come out of the system, and thankfully we’ve never had to figure out how to come back into the system, but when you’re getting up to day 12, day 13, you think we’re coming up to day 14 and you think OK her services are going to get cut off, now the transition to home looks very, very different….”*

### Theme 2: Positive experiences during care transitions

Participants shared their positive experiences when navigating transitions between services as indicated in sub-themes of coordination of care and the nurse involvement in care coordination.

#### Coordination of care

This sub-theme was defined as coordination between healthcare providers in different sectors of the healthcare system. P3 was able to describe effective communications between the providers; he said: “*they had all my files from the [hospital] and they knew I was diabetic and everything else…”* And F7 explained the successful discharge information provided to the family physician, as follows: *“all the information [was] all sent to the family doctor. Prescription changes and …what he was sent to the hospital for and everything like that. It’s all sent to the doctor and… when we go to him he’s already got all that stuff.”*

#### Nurse involvement in care coordination

Participants reported that the nurse played a significant role in facilitating their care transition. For example, PF4 explained that *“The nurses took care of making sure that the ambulance came, and the doctor reached out to the doctor in the emergency.”* P2 said: *“happy that I’m getting this care, because if not I would be falling in between the cracks*.” P9 expressed their satisfaction about the communication from nurses, stating: *“both my wife and I were in sync.”* P1 said: *“They got him [son] more involved, so he really knows what’s going on.”* PF5 explained: *“the [home care services] nurse came in and said, ‘This is what’s going to happen,’ and it’s just in the implementation and I was able to communicate with her when things were not quite there.”*

### Theme 3: Accessing community services and resources

Another key theme during care transitions was accessing community services and resources. Sub-themes were responsive and personalized care, home support, and knowledge of resources.

### Responsive and personalized care

P9 reported benefits from living in a small, rural community, such as being able to access a family doctor quickly. *“My friends in [the city] could not phone in the same day and say I’m coming in at 3 o’clock.”* P9 also reported receiving more personalized care from the pharmacist, *“we’re very lucky we have a good pharmacy; we get a lot of personal service that you wouldn’t get out in the city. I think it’s a small community they know him; he’s been going for 30 years.”*

### Home support

Some participants described that they were able to access local home support services after discharge from hospital. P2 described her experience with the community nurse’s support following discharge from the hospital. *“When [the Health Link nurse] told her, if you don’t feel good, call me. If I can I will drop what I’m doing and I will come over right away.”* P9 said that when *“the nurse came here, he followed right through, I have nothing negative to say.”* PF4 described access to the Rapid Response program, which helped to support the transition between services. *“In the Going Home program you had that young girl come and she did the house cleaning for a couple of hours for a couple of weeks. That was helpful. Again because she is a [home care services’] client, the rapid response nurse came in when she was released from the hospital.”*

### Knowledge of resources

**Fig. 3:**
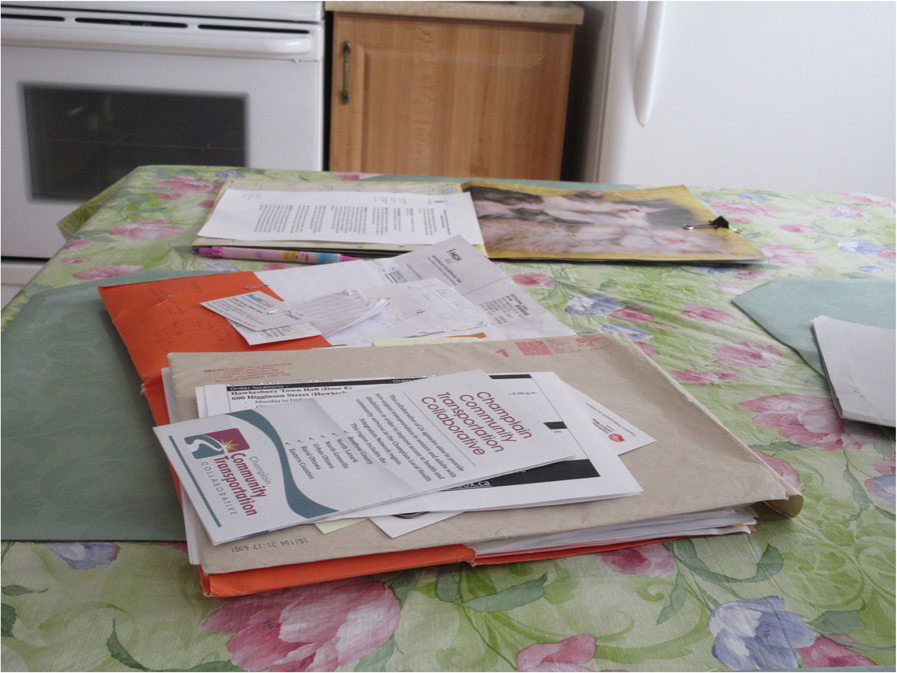
Documents of community resources available

**Fig. 4:**
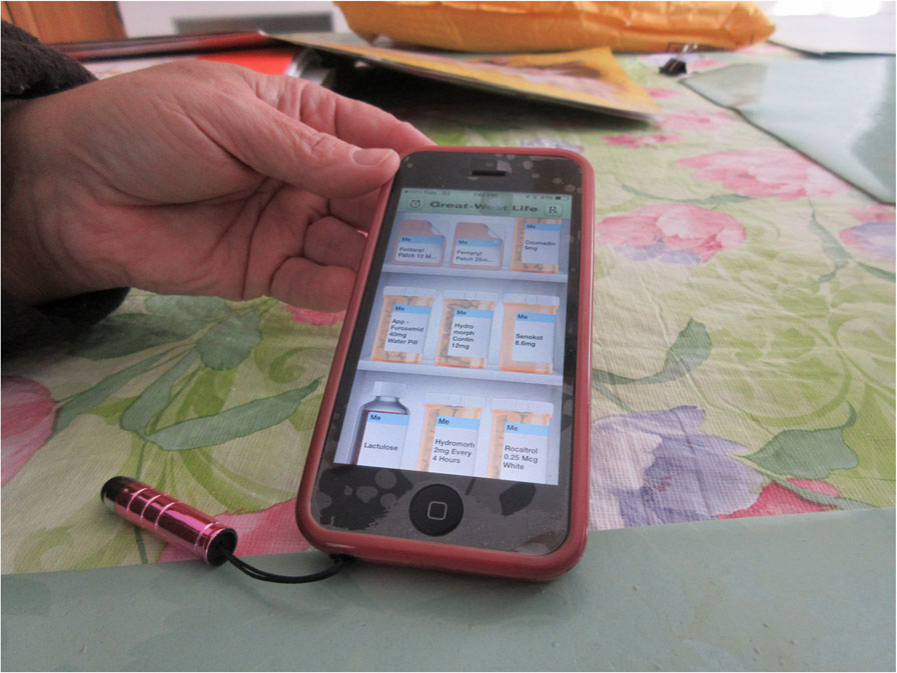
Mobile app to track medications

P2 showed the volume of information received by the Health Link about community resources available (Fig. 3), and described that: *“… [the Health Link providers] were helpful, and they gave me names of people for my husband and myself so if we cannot do some of the work around here, they gave me names of people I can contact. For that it’s been perfect, I managed to get a whole bunch of stuff and resources I didn’t know that I had and I didn’t know that Ontario offered either.”* P2 also described the mobile app she was made aware of how to track her medications (Fig. 4), and explained: “*That’s good, because when you go to a doctor for an older person this app is easy to get, you have to keep it updated, which I do, but it has all the medication, the quantities. The last time when I went to the doctor, I forgot my folder, and then I had it in my phone but I forgot I had it. With the list of names in phone and doctors and their numbers. I also have notes in the phone, and reminders. All my appointments are listed…So everything done, every appointment listed, but when I moved here I didn’t keep track. I write the time the appointment and what it was for. It keeps it all organized.”*

### Theme 4: Challenges with follow-up care

Participants described having multiple specialists involved in their care, based on their complex needs, which resulted in many logistical issues to manage, including travelling to multiple appointments, and trying to coordinate the different aspects of care. Sub-themes included: difficulties booking follow-up appointment, challenges getting to follow-up appointments, timely access to primary care, lack of coordination of care, and adverse events post-discharge.

#### Difficulties booking follow-up appointment

PF4 explained the challenge with booking a follow-up appointment with a specialist, saying: *“I was given a card and was told to call, unfortunately I called and was given the wrong number to call.”* And further explained: “*The number I was given said they didn’t do it, and said I had to call up the neurologist directly. So I called the neurologist directly, she was away because it was over the Christmas holidays. And when I finally got through to her, she said absolutely the ones who I had spoken to [originally] were the ones who should have booked the appointment.”* P2 explained: *“I have to ask what happened to the bariatric doctor. He said he sent a requisition form, but I haven’t heard a thing from them.”*

#### Challenges getting to follow-up appointments

F6 explained her challenges with bringing her family member to physician appointments: *“how am I supposed to take a 94-year old lady downtown to [an appointment]… park the car…and it’s right near the Byward Market…Better off taking a taxi, honest to god. And I never took her… I can’t get her out of the house for anything unless you’re taking her by, you know, ambulance, god forbid.”* P1 said: *“I called her [the doctor’s office] and told them I couldn’t go in because I just couldn’t drive in, I couldn’t even get a taxi to take me in because I couldn’t even get dressed.”* PF5 described: *“if there was someone who was a primary care person that minded the medical side of things at a more advanced level, that would be helpful. Because she’s got congestive heart failure, how are you going to monitor and how are you going to signal what she needs.”*

#### Timely access to primary care

Participants identified travelling long distances to medical centres for follow-up appointments as problematic. PF5, whose mother lives in an assisted living facility found it difficult to access the family doctor outside of their usual visiting time, and felt that a timely visit from a family doctor could have prevented her mother’s readmissions to the hospital. *“Two admissions could have been prevented if there was some other strategy, you can’t always rely on the primary care doctor here to see her once a week or she could have something in between the weeks.”* P1 described the challenge with having timely visits with her family doctor. *“The secretary [in March]… said she was changing the date to the end of April, I said the end of April I’ll be dead.”*

#### Lack of coordination of care

P2 felt that “*if they had one doctor who would actually communicate with the other doctors and say…something has to change, your prescriptions are not right, let’s just talk together amongst ourselves and let’s see if we can fix that little glitch.”* P2 also explained that: “*when I was in the hospital, I had asked them to give me a photocopy of all the tests I had done and they said that they wouldn’t give it to me. Normally when I go to a hospital, I ask for the photocopies of what’s been done, because as I said, I keep a record of everything, of all of my medical problems.”*

#### Adverse events post-discharge

F8 described her mother’s complication post-discharge, and the difficulty in receiving the appropriate treatment in a timely manner. F8 explained: “*she started to have swollen legs and so I said, you know, you just have to look after her, you know, physical needs at least, you know, and … so they did… they did say they’d call the doctor. Well, they called her family doctor instead of the surgeon. Family doctor knew nothing. Sent a prescription for I guess a water pill and, you know, ordered an x-ray and I mean but she was out of it. You know, she did what she could but, you know, they should have called the surgeon.”*

### Theme 5: Lack of meaningful engagement during discharge planning

Lack of meaningful engagement during care transitions included the sub-themes: lack of involvement in discharge planning and not prepared for discharge.

#### Lack of involvement in discharge planning

Some participants felt that they were not empowered in a strategic way particularly around the discharge planning. P2 explained: *“I think as you’re leaving the hospital there’s a little bit of a thing there that happens when you’re being discharged they don’t quite exactly explain the medications and the doctor that should follow you.”* F6 explained the lack of opportunity to seek further information: *“so we get there, a psychiatrist comes in, gives me paperwork, says these are the pills I put her on, and everything is glorious and wonderful and he leaves. So I don’t get much time with him at all. And I get it that they’re all busy but that’s ridiculous.”* F8 explained the lack of information received about her mother’s discharge instructions: *“No, I didn’t see that care plan. No. Certainly all the medications they wanted her to take, lots of painkiller, you know…”.*

#### Not prepared for discharge

Participants described having little notice of discharge, meaning there was little time to plan services and the transition home. P1 said: *“I can’t get anything planned at home. I will come home to a dirty house, I mean, doctors don’t tell you nothing, you can go home this afternoon, OK?”* PF5 explained: *“They just said I was going home, I was glad. [But] no preparation, you know.”* Participants also described poor teaching around discharge plans as an issue, increasing the anxiety during transitions. F7 explained: *“I didn’t know what to do. I was near tears at one point because I just didn’t know what to do…and then I got a little agitated with the doctor because, you know, they’re telling me one thing and then something else and it just… emotionally, it… I didn’t know whether to cry or scream.”*

### Theme 6: Presence of systemic barriers in care transitions

Presence of systemic barriers in care transitions included sub-themes of limited access to resources, and provider-centered care.

#### Limited access to resources

P1 was admitted to hospital a number of times because she was not in a position to pay for her own oxygen supply, and was not deemed unwell enough to have provincially funded oxygen on prescription. This resulted in what she perceived to be unnecessary admissions to hospital resulting in multiple transitions between inpatient and community services. *“I was in for two weeks and out for two weeks, then back in for two weeks and out for two weeks. Because they wouldn’t give me the oxygen I needed… your blood count has to be so low before you are allowed to get it for free, so I couldn’t have afforded to pay for this, it’s an enormous price.”* F6 explained that she would have benefited from knowing the resources available to her. She said: *“so just to let you know, I am not well versed on everything that might be available to me, as a person caregiving an elderly.”* P2 explained her difficulty in being reimbursed by the government for her prescription: *“I couldn’t get the Vicomyacin, and I was running around as only one pharmacy had it in [the city]. I was finally being able to get it but then I had to fight with [the ministry] to get my money back which shouldn’t happen*.”

#### Provider-centered care

P3 explained that the care received was provider-centered: *“But they couldn’t do it on the Friday because it was the long weekend.”* P2 explained our health system as “siloed”: “*when I go to my kidney doctor, you’re going there, it’s only for the kidneys, don’t talk about anything else, we’re only here for the kidneys, and you know that’s where I think there’s just something more to bridge that together.”* F8 described the multiple appointments they experienced: “*So there were eleven pre-op appointments. A few of them were bunched…like the occupational therapist and the physiotherapist we saw and one other… the nurse at the hospital we saw on the same day. But, you know, then she had to go a hematologist because she was on a blood thinner and they wanted to make sure that, you know, it’s going to be okay for surgery… and again a cardiac person and again, you know, a blood… the bloodwork and x-rays and so on. So you know, you’re worn out by the time the surgery comes and to have to fight just to get simple, simple care. She just needed somebody to look in on her, you know.”*

## Discussion

This research study aimed at using participatory visual narrative methods to actively engage older adults and families in detailed exploration of their experiences of managing multiple chronic conditions while transitioning across the health care system. The themes identified in this study included: the importance of active involvement in managing care transitions, positive experiences during care transitions, accessing community services and resources, challenges with follow-up care, lack of meaningful engagement during discharge planning as well as the presence of systemic barriers in care transitions.

While our small study sample was confined to one geographic area within one Canadian province, it is notable how convergent our findings are with some other larger studies of older adult home care clients and cargivers managing complex health conditions. Specifically, we can map one or more elements of every theme in our study findings to key findings in a four province study on medication safety for older adult home care clients and their families [34]. Similar to our participants, clients, caregivers and providers in this pan-Canadian study expressed complex challenges with their health conditions, personal circumstances, sources of support, and systemic barriers that affected their ability to safely manage and avoid preventable harms. In addition, similar to our findings, participants in the study by Lang and colleagues cited multiple examples of inadequate client and family engagement in care planning and care transitions; poorly coordinated care; the need for continual caregiver advocacy; households devising their own tracking and communication strategies to compensate for system deficiencies; and the value of system navigators where available to reduce communication gaps and improve the integration of care.

Other studies also confirm the validity of our concerns about communication gaps and uncoordinated care for older adults managing complex co-morbidities across poorly integrated health systems [34–37]. Furthermore, building confluence of findings around the importance of actively engaging older adults and their caregivers as care partners warrants focussed attention [7, 38]. This convergence of findings across studies suggest at least three areas for improvement specific to older adults managing chronic conditions during care transitions, which are described below.

1. Strengthening support for person- and family-centered integrated care

Older adults and families in our study described the nurse as the central hub that links them to healthcare providers and community resources. In addition to the medical needs of this population, it was clear that appropriate assessment and mechanisms to support patients in the broader context of health, which includes economic, access, and social issues/barriers are needed. Older adults and families often do not know who to contact to obtain support. As one participant mentioned, the care is often focused on the medical issue, and thus providing a very “siloed” system. One consideration to facilitate better integration is assigning a primary provider as the central hub to be the link during the patient’s transitions across the health care system. Previous studies show that such system navigator roles have reduced readmissions to hospital, and return visits to the emergency department [39–44].

2) Engaging older adults and families in their care management and care transitions in meaningful ways

Our study indicated that older adults and family members who were actively involved in their care were better able to navigate their journey through the health care system. This is consistent with other studies [34, 45, 46]. Emerging research is focused on further engaging patients and families in their care [47–49]. Further work is needed to develop personalized care which provides individualized access to relevant resources in order to better manage not only single but multiple chronic conditions. One promising option would be to explore the use of technology to provide a personalized care plan approach to ensure safe, effective and person- and family-centered care transitions. The development of new technologies for personalized care could potentially help to facilitate and manage multiple chronic conditions while navigating our complex healthcare system, and is empowering to the patient and their families [50].

3) Providing adequate support/resources for family members and informal caregivers

It is clear from this research and from other studies [13, 51] that family members and informal caregivers take an active role in helping patients during their care transition experiences. Further resources are needed to better support family members who play an active role in care. It is important however to assess a patient’s social context to determine if further support is needed, where there is or is not the presence of informal caregivers and family members who help to support the patient.

There are strengths and limitations to our study findings. First, our approach to engage older adults with multiple chronic conditions, and families, to capture their ‘voice’, their experiences during care transitions was challenging due to recent changes with local home care services. These changes to improve coordination of services and planning are a result of new provincial legislation to replace the current home care service agency model, and expand the role of Local Health Integration Networks to oversee home and community care. This decision resulted in some difficulty in accessing participants for our study. A strength to the study is that we used a novel method - participatory visual narrative methods - to gain in-depth perspectives from the participants.

## Conclusion

Our study findings add support to other research findings. They provide perspectives of older adults and their families about a lack of meaningful engagement during discharge planning, as well as the overall importance of their active involvement during care transitions. Future research should emphasize the perspective of older adults and their families in the co-design and the implementation of person- and family-centered evidence-based care transition interventions.
